# Bone Marrow Stem Cell as a Potential Treatment for Diabetes

**DOI:** 10.1155/2013/329596

**Published:** 2013-04-10

**Authors:** Ming Li, Susumu Ikehara

**Affiliations:** Department of Stem Cell Disorders, Kansai Medical University, Moriguchi, Osaka 570-8506, Japan

## Abstract

Diabetes mellitus (DM) is a group of metabolic diseases in which a person has high blood glucose levels resulting from defects in insulin secretion and insulin action. The chronic hyperglycemia damages the eyes, kidneys, nerves, heart, and blood vessels. Curative therapies mainly include diet, insulin, and oral hypoglycemic agents. However, these therapies fail to maintain blood glucose levels in the normal range all the time. Although pancreas or islet-cell transplantation achieves better glucose control, a major obstacle is the shortage of donor organs. Recently, research has focused on stem cells which can be classified into embryonic stem cells (ESCs) and tissue stem cells (TSCs) to generate functional **β** cells. TSCs include the bone-marrow-, liver-, and pancreas-derived stem cells. In this review, we focus on treatment using bone marrow stem cells for type 1 and 2 DM.

## 1. Introduction

Diabetes mellitus (DM) is a devastating disease [1] that includes 2 main types: type 1 and type 2 DM. DM therapies mainly include diet, insulin, oral hypoglycemic agents, and pancreas or islet-cell transplantation. Exogenous insulin replacement has been the primary therapeutic technique for controlling plasma glucose levels. However, because of the shortage of donor organs, recent research has focused on stem cells, including pancreatic stem cells, hepatic stem cells, bone marrow stem cells (BMSCs), induced pluripotent stem cells (iPS), and embryonic stem cells (ESCs) to generate *β* cells for DM treatment [2]. BM is an invaluable source of adult pluripotent stem cells, and this review focuses on the treatment of DM using BMSCs.

## 2. Pathophysiology of Type 1 DM

Type 1 DM is a T-cell-mediated autoimmune disease accompanying lymphocytic infiltration of the pancreatic islets. The nonobese diabetic (NOD) mouse is a spontaneous mouse model of type 1 DM and involves many of the same autoantigens targeted by human T cells [3, 4]. BM-derived T-cell precursors go to the thymus to differentiate into mature T cells via positive and negative selection. Thymocytes expressing TCRs recognize self-antigen-MHC complexes with high affinity/avidity undergoing central deletion. In contrast, thymocytes expressing low-affinity TCRs for the same complexes differentiate into mature T cells and populate the peripheral lymphoid organs where they become available for recognizing foreign antigens [5]. Autoreactive T cells, with their relatively high avidity and pathogenic potential, can escape thymocyte negative selection and elicit autoimmunity in the absence of adequate peripheral regulation [6]. Approximately 20% of individuals with spontaneous mutation of autoimmune gene Aire develop type 1 DM with other autoimmune diseases, which is thought to reflect their inability to select against islet antigen reactivity during T-cell development [7]. Insulin is an Aire-regulated islet protein ectopically expressed in the thymic medullary epithelial cells. Insulin was detected in all thymus tissues examined and class III VNTR alleles were associated with a 2-to-3-fold higher INS mRNA level than class I. It has been proposed that higher levels of thymic INS expression, which facilitates the induction of immune tolerance, are a mechanism for the dominant protective effect of class III alleles [8]. 

In the NOD mouse model, mice that are genetically deficient in B cells from birth develop a very low incidence of diabetes [9, 10]. One report has suggested that depleting B cells delayed and/or reduced the onset of diabetes and reversed diabetes in over one-third of mice and that B cells are among the regulatory populations [11].

## 3. BMSC Therapies for Type 1 DM

Insulin injection is the standard therapy for type 1 DM. Unfortunately, however, exogenous insulin injection cannot mimic insulin secretion from normal *β* cells when blood glucose changes all the time. Thus, how to generate new *β* cells is an important approach in the treatment of type 1 DM. BM-, liver-, and pancreas-derived stem cells, iPS cells, and ESCs can differentiate into *β* cells. Hepatic stem cells are excellent candidates for generating *β*-cell surrogates because the liver and pancreas are both differentiated from endoderm during development, and liver stem cells expressing duodenum homeobox protein-1 long-term exhibited similar profiles for the expression of genes related to pancreatic development and *β*-cell function and reversed hyperglycemia in diabetic mice [12]. The acinar cells in the pancreas produce digestive enzymes and duct cells that form the afferent system to the duodenum. Adult mouse pancreas contains islet cell progenitors, which differentiate into *β* cells after partial duct ligation. This cell population represents an obvious target for therapeutic regeneration of *β* cells in DM [13]. iPS cell transplantation corrected hyperglycemia in two mouse models of types 1 and 2 DM [14]. Although ESCs are pluripotent stem cells and can generate insulin-positive cells *in vitro* [15], their differentiation *in vitro* cannot be controlled [16].

BM is the flexible tissue found in the interior of bones, and includes hematopoietic stem cells (HSCs), mesenchymal stem cells (MSCs), and endothelial progenitor cells (EPCs). HSCs give rise to red blood cells, platelets, monocytes, granulocytes, and lymphocytes; MSCs can differentiate into myogenic, osteogenic, chondrogenic, and adipogenic lineages [17, 18]. EPCs are a population of rare cells that circulate in the blood with the ability to differentiate into the endothelial cells that make up the lining of blood vessels [19]. BM cells (BMCs) have the ability to differentiate *in vivo* into functionally competent *β* cells [20]. 

NOD mice that received transplanted BALB/c nu/nu bone marrow cells displayed normal T- and B-cell functions, and newly developed T cells in the allogeneic bone marrow recipients were tolerant to cells with both donor- and host-type major histocompatibility complex determinants. These results suggest that allogenic bone marrow transplantation (ABMT) might contribute to the prevention of islet destruction and to the restoration of self-tolerance [21]. However, ABMT alone could not be used to treat overt diabetes in NOD mice whose islets had been completely destroyed. One report has demonstrated that BMT promotes *β*-cell regeneration after acute injury through BM mobilization [22]. NOD mice showed a reduction of the glycosuria and a normal response in the glucose-tolerance test after the transplantation of bone marrow plus newborn pancreas [23, 24]. The transplantation of pancreatic islets from two MHC-disparate donors was achieved in combination with IBM-BMT, resulting in the improvement of blood glucose levels and the amelioration of streptozotocin-induced DM in rats [25]. SDF-1 could potentially be used to improve the homing of stem cells and *β*-cell regeneration, and it improves glycemia and insulin production in diabetic mice [26]. The transplantation of insulin-producing cells from adult hBMSCs into nude diabetic mice resulted in the control of their diabetic status for 3 months [27]. Recent reports have demonstrated that human bone marrow MSCs (hBMMSCs) improved blood glucose control and ameliorated diabetes in animal models [28–31]. One report has described the possible benefit of hBMMSCs for the treatment of insulin-dependent diabetes and gives new insight into the mechanism of *β*-cell recovery after injury mediated by hBMMSC therapy [32].

Hyperglycemia affects EPC function by generating diffused endothelial damage, microvascular remodeling, and a reduction in the number of c-kit+Scal-1+ cells [33]. Reports have shown that adverse metabolic stress factors in type 1 DM are associated with reduced EPC numbers and angiogenicity [34]. Type 2 DM may alter EPC biology in processes critical for new blood vessel growth and may identify a population at high risk for morbidity and mortality after vascular occlusive events [35]. EPCs-mediated neovascularization of the pancreas could in principle be exploited to facilitate the recovery of nonterminally injured *β*-cells or to improve the survival and/or function of islet allografts [36].

BMMSCs can not only promote endogenous angiogenesis [37] but also directly differentiate into smooth muscle [38] and endothelial cell [39] phenotypes *in vitro* and into functional vascular structures [40]. They contribute to myocardial recovery after injury *in vivo *[41]. MSCs have been shown to significantly suppress *β* cell-specific T-cell proliferation in the pancreas. Moreover, the increase in *β*-cell regeneration may be partly attributable to the protective qualities of MSCs, which shield newly formed *β* cells from destruction by T cells and thereby overcoming the inherent autoimmune pathology associated with type 1 DM [29]. BMCs have already been used in Phases I to III of clinical trials (NCT00821899, NCT01143168, NCT01157403, and NCT00465478) for the treatment of type 1 DM, as has been listed in the Clinical Trails.gov registry (update to August 2012).

## 4. Pathophysiology of Type 2 DM

Metabolic and immune systems are among the most fundamental requirements for survival. Obesity, insulin resistance, and type 2 DM are closely associated with chronic inflammation [42]. Genetic and environmental factors are involved in the development of type 2 DM, which shows relative insulin deficiency because of insulin resistance [43]. Adipocytes regulate and mediate inflammatory cytokines such as tumor necrosis factor-*α* (TNF*α*) and IL-6, which, respectively, inhibit or enhance each other, contributing to insulin resistance [44]. The basic mechanism of type 2 DM is that *β* cells fail to compensate for the effect of insulin resistance [45, 46].

Some youths with a clinical diagnosis of type 2 DM show evidence of islet-cell autoimmunity, with autoantibodies including islet-cell antibodies (ICA), glutamic acid decarboxylase (GAD), islet-autoantibodies-(IA-) 2, and insulin antibodies [47, 48]. Obese youths with a clinical diagnosis of type 2 DM may show evidence of islet autoimmunity contributing to insulin deficiency [49]. 

## 5. BMSC Therapies for Type 2 DM

We previously showed that KK-Ay mice, a type 2 DM model reconstituted with KK-Ay bone marrow cells, showed glycosuria, hyperinsulinemia, and hyperlipidemia. However, KK-Ay mice showed improved serum insulin and lipid levels 4 months after BMT from normal BALB/c mice [50]. A previous report suggested that the transplantation of BMMSCs via intra-bone-marrow-BMT (IBM-BMT) in conjunction with the induction of HO-1 could eradicate type 2 DM. The beneficial effect of HO-1 induction further suggests that the abnormality in endothelial progenitor cells is due to a MSC-stromal cell disorder exacerbated by oxidative stress and decreases in adiponectin [51]. Autologous BM-derived rat MSCs promote PDX-1 and insulin expression in the islets and alter T-cell cytokine patterns [52]. Combined therapy of intrapancreatic autologous stem cell infusion and hyperbaric oxygen treatment can improve metabolic control and reduce insulin requirements in patients with type 2 DM [53]. 

The leptin receptor-deficient db/db mouse, a type 2 DM mouse model, exhibits severe hereditary obesity [54] and displays hormonal imbalances and hematolymphoid defects [55, 56]. Db/db mice exhibit a marked reduction in the size and cellularity of the thymus [57, 58]. We have reported that, in db/db mice, increased insulin sensitivity and decreased blood glucose levels result from normalizing any imbalance in lymphocyte subsets by IBM-BMT+ thymus transplantation (TT). The novel effects of IBM-BMT+TT are that this combination induces adiponectin secretion, followed by enhanced pLKB1-AKT-AMPK crosstalk, signaling pathway, insulin phosphorylation, and also HO-1 [59]. Moreover, IBM-BMT+TT has been proved to upregulate the expression of HO-1, peNOS, and pAKT, while decreasing iNOS levels in the kidney of db/db mice [60]. BMCs have already been used in Phases I to III of clinical trials (NCT00465478, NCT00644241, NCT01142050, NCT01677013, and NCT01759823) for the treatment of type 2 DM, as has been listed in the Clinical Trails.gov registry (update to August 2012). Combined therapy of intrapancreatic BMT and hyperbaric oxygen treatment can improve glucose control and reduce the dose of insulin and/or oral hypoglycemic drugs in type 2 DM patients, even if only to improve pancreatic *β*-cell function transiently [61].

## 6. Conclusion

There are problems which have a tendency to form teratomas and the ethical debate revolving around their derivation on the use of ESCs. iPS cells have been shown to have an embryonic-like pluripotent state that enables the development of an unlimited source of any type of human cell. However, the safety of using iPSCs needs to be proven in clinical trials. In contrast, BMSCs are an invaluable source of adult of pluripotent stem cells and have already been used in Phases I to IV of clinical trials for the treatment of type 1 and 2 DM. BMSCs are thus a potential agent for the treatment of DM.

## References

[1] American Diabetes Association (2004). Diagnosis and classification of diabetes mellitus. *Diabetes Care*.

[2] Hussain MA, Theise ND (2004). Stem-cell therapy for diabetes mellitus. *The Lancet*.

[3] Delovitch TL, Singh B (1997). The nonobese diabetic mouse as a model of autoimmune diabetes: immune dysregulation gets the NOD. *Immunity*.

[4] Atkinson MA, Leiter EH (1999). The NOD mouse model of type 1 diabetes: as good as it gets?. *Nature Medicine*.

[5] Marrack P, Parker DC (1994). A little of what you fancy. *Nature*.

[6] Han B, Serra P, Yamanouchi J (2005). Developmental control of CD8^+^ T cell-avidity maturation in autoimmune diabetes. *Journal of Clinical Investigation*.

[7] Gardner JM, Fletcher AL, Anderson MS, Turley SJ (2009). AIRE in the thymus and beyond. *Current Opinion in Immunology*.

[8] Vafiadis P, Bennett ST, Todd JA (1997). Insulin expression in human thymus is modulated by INS VNTR alleles at the IDDM2 locus. *Nature Genetics*.

[9] Serreze DV, Chapman HD, Varnum DS (1996). B lymphocytes are essential for the initiation of T cell-mediated autoimmune diabetes: analysis of a new “speed congenic” stock of NOD.Ig mu null mice. *Journal of Experimental Medicine*.

[10] Akashi M, Nagafuchi S, Anzai K (1997). Direct evidence for the contribution of B cells to the progression of insulitis and the development of diabetes in non-obese diabetic mice. *International Immunology*.

[11] Hu CY, Rodriguez-Pinto D, Du W (2007). Treatment with CD20-specific antibody prevents and reverses autoimmune diabetes in mice. *Journal of Clinical Investigation*.

[12] Yang LJ (2006). Liver stem cell-derived *β*-cell surrogates for treatment of type 1 diabetes. *Autoimmunity Reviews*.

[13] Xu X, D’Hoker J, Stangé G (2008). *β* cells can be generated from endogenous progenitors in injured adult mouse pancreas. *Cell*.

[14] Alipio Z, Liao W, Roemer EJ (2010). Reversal of hyperglycemia in diabetic mouse models using induced-pluripotent stem (iPS)-derived pancreatic *β*-like cells. *Proceedings of the National Academy of Sciences of the United States of America*.

[15] Segev H, Fishman B, Ziskind A, Shulman M, Itskovitz-Eldor J (2004). Differentiation of human embryonic stem cells into insulin-producing clusters. *Stem Cells*.

[16] Brolén GKC, Heins N, Edsbagge J, Semb H (2005). Signals from the embryonic mouse pancreas induce differentiation of human embryonic stem cells into insulin-producing *β*-cell-like cells. *Diabetes*.

[17] Pittenger MF, Mackay AM, Beck SC (1999). Multilineage potential of adult human mesenchymal stem cells. *Science*.

[18] Colter DC, Class R, DiGirolamo CM, Prockop DJ (2000). Rapid expansion of recycling stem cells in cultures of plastic-adherent cells from human bone marrow. *Proceedings of the National Academy of Sciences of the United States of America*.

[19] Khoo CP, Pozzilli P, Alison MR (2008). Endothelial progenitor cells and their potential therapeutic applications. *Regenerative Medicine*.

[20] Ianus A, Holz GG, Theise ND, Hussain MA (2003). In vivo derivation of glucose-competent pancreatic endocrine cells from bone marrow without evidence of cell fusion. *Journal of Clinical Investigation*.

[21] Ikehara S, Ohtsuki H, Good RA (1985). Prevention of type I diabetes in nonobese diabetic mice by allogeneic bone marrow transplantation. *Proceedings of the National Academy of Sciences of the United States of America*.

[22] Hasegawa Y, Ogihara T, Yamada T (2007). Bone marrow (BM) transplantation promotes *β*-cell regeneration after acute injury through BM cell mobilization. *Endocrinology*.

[23] Yasumizu R, Sugiura K, Iwai H (1987). Treatment of type 1 diabetes mellitus in non-obese diabetic mice by transplantation of allogeneic bone marrow and pancreatic tissue. *Proceedings of the National Academy of Sciences of the United States of America*.

[24] Iwai H, Yasumizu R, Sugiura K (1987). Successful pancreatic allografts in combination with bone marrow transplantation in mice. *Immunology*.

[25] Taira M, Inaba M, Takada K (2005). Treatment of streptozotocin-induced diabetes mellitus in rats by transplantation of islet cells from two major histocompatibility complex disparate rats in combination with intra bone marrow injection of allogenic bone marrow cells. *Transplantation*.

[26] Cheng H, Zhang YC, Wolfe S (2011). Combinatorial treatment of bone marrow stem cells and stromal cell-derived factor 1 improves glycemia and insulin production in diabetic mice. *Molecular and Cellular Endocrinology*.

[27] Gabr MM, Zakaria MM, Refaie AF Insulin-producing cells from adult human bone marrow mesenchymal stem cells control streptozotocin-induced diabetes in nude mice.

[28] Lee RH, Seo MJ, Reger RL (2006). Multipotent stromal cells from human marrow home to and promote repair of pancreatic islets and renal glomeruli in diabetic NOD/scid mice. *Proceedings of the National Academy of Sciences of the United States of America*.

[29] Urbán VS, Kiss J, Kovács J (2008). Mesenchymal stem cells cooperate with bone marrow cells in therapy of diabetes. *Stem Cells*.

[30] Fiorina P, Jurewicz M, Augello A (2009). Immunomodulatory function of bone marrow-derived mesenchymal stem cells in experimental autoimmune type 1 diabetes. *Journal of Immunology*.

[31] Ezquer F, Ezquer M, Simon V, Conget P (2011). The antidiabetic effect of MSCs is not impaired by insulin prophylaxis and is not improved by a second dose of cells. *PLoS ONE*.

[32] Milanesi A, Lee JW, Li Z (2012). beta-Cell regeneration mediated by human bone marrow mesenchymal stem cells. *PLoS ONE*.

[33] Oikawa A, Siragusa M, Quaini F (2010). Diabetes mellitus induces bone marrow microangiopathy. *Arteriosclerosis, Thrombosis, and Vascular Biology*.

[34] Loomans CJM, De Koning EJP, Staal FJT (2004). Endothelial progenitor cell dysfunction: a novel concept in the pathogenesis of vascular complications of type 1 diabetes. *Diabetes*.

[35] Tepper OM, Galiano RD, Capla JM (2002). Human endothelial progenitor cells from type II diabetics exhibit impaired proliferation, adhesion, and incorporation into vascular structures. *Circulation*.

[36] Mathews V, Hanson PT, Ford E, Fujita J, Polonsky KS, Graubert TA (2004). Recruitment of bone marrow-derived endothelial cells to sites of pancreatic beta-cell injury. *Diabetes*.

[37] Duffy GP, Ahsan T, O’Brien T, Barry F, Nerem RM (2009). Bone marrow-derived mesenchymal stem cells promote angiogenic processes in a time- and dose-dependent manner in vitro. *Tissue Engineering A*.

[38] Tamama K, Sen CK, Wells A (2008). Differentiation of bone marrow mesenchymal stem cells into the smooth muscle lineage by blocking ERK/MAPK signaling pathway. *Stem Cells and Development*.

[39] Yue WM, Liu W, Bi YW (2008). Mesenchymal stem cells differentiate into an endothelial phenotype, reduce neointimal formation, and enhance endothelial function in a rat vein grafting model. *Stem Cells and Development*.

[40] Gong Z, Niklason LE (2008). Small-diameter human vessel wall engineered from bone marrow-derived mesenchymal stem cells (hMSCs). *The FASEB Journal*.

[41] Quevedo HC, Hatzistergos KE, Oskouei BN (2009). Allogeneic mesenchymal stem cells restore cardiac function in chronic ischemic cardiomyopathy via trilineage differentiating capacity. *Proceedings of the National Academy of Sciences of the United States of America*.

[42] Wellen KE, Hotamisligil GS (2005). Inflammation, stress, and diabetes. *Journal of Clinical Investigation*.

[43] Hotamisligil GS (2006). Inflammation and metabolic disorders. *Nature*.

[44] Suganami T, Nishida J, Ogawa Y (2005). A paracrine loop between adipocytes and macrophages aggravates inflammatory changes: role of free fatty acids and tumor necrosis factor *α*. *Arteriosclerosis, Thrombosis, and Vascular Biology*.

[45] Fonseca VA (2009). Defining and characterizing the progression of type 2 diabetes. *Diabetes Care*.

[46] Hanley SC, Austin E, Assouline-Thomas B (2010). *β*-cell mass dynamics and islet cell plasticity in human type 2 diabetes. *Endocrinology*.

[47] Hathout EH, Thomas W, El-Shahawy M, Nahab F, Mace JW (2001). Diabetic autoimmune markers in children and adolescents with type 2 diabetes. *Pediatrics*.

[48] Gilliam LK, Brooks-Worrell BM, Palmer JP, Greenbaum CJ, Pihoker C (2005). Autoimmunity and clinical course in children with type 1, type 2, and type 1.5 diabetes. *Journal of Autoimmunity*.

[49] Klingensmith GJ, Pyle L, Arslanian S (2010). The presence of GAD and IA-2 antibodies in youth with a type 2 diabetes phenotype: results from the TODAY study. *Diabetes Care*.

[50] Than S, Ishida H, Inaba M (1992). Bone marrow transplantation as a strategy for treatment of non-insulin- dependent diabetes mellitus in KK-Ay mice. *Journal of Experimental Medicine*.

[51] Abraham NG, Li M, Vanella L, Peterson SJ, Ikehara S, Asprinio D (2008). Bone marrow stem cell transplant into intra-bone cavity prevents type 2 diabetes: role of heme oxygenase-adiponectin. *Journal of Autoimmunity*.

[52] Boumaza I, Srinivasan S, Witt WT (2009). Autologous bone marrow-derived rat mesenchymal stem cells promote PDX-1 and insulin expression in the islets, alter T cell cytokine pattern and preserve regulatory T cells in the periphery and induce sustained normoglycemia. *Journal of Autoimmunity*.

[53] Estrada EJ, Valacchi F, Nicora E (2008). Combined treatment of intrapancreatic autologous bone marrow stem cells and hyperbaric oxygen in type 2 diabetes mellitus. *Cell Transplantation*.

[54] Chua SC, Chung WK, Wu-Peng XS (1996). Phenotypes of mouse diabetes and rat fatty due to mutations in the OB (leptin) receptor. *Science*.

[55] Fantuzzi G, Faggioni R (2000). Leptin in the regulation of immunity, inflammation, and hematopoiesis. *Journal of Leukocyte Biology*.

[56] Matarese G, Moschos S, Mantzoros CS (2005). Leptin in immunology. *Journal of Immunology*.

[57] Fernandes G, Handwerger BS, Yunis EJ, Brown DM (1978). Immune response in the mutant diabetic C57BL/Ks-db+ mouse. Discrepancies between in vitro and in vivo immunological assays. *Journal of Clinical Investigation*.

[58] Kimura M, Tanaka SI, Isoda F, Sekigawa KI, Yamakawa T, Sekihara H (1998). T lymphopenia in obese diabetic (db/db) mice is non-selective and thymus independent. *Life Sciences*.

[59] Li M, Abraham NG, Vanella L (2010). Successful modulation of type 2 diabetes in db/db mice with intra-bone marrow-bone marrow transplantation plus concurrent thymic transplantation. *Journal of Autoimmunity*.

[60] Li M, Vanella L, Zhang Y (2012). Stem cell transplantation increases antioxidant effects in diabetic mice. *International Journal of Biological Sciences*.

[61] Wang L, Zhao S, Mao H (2011). Autologous bone marrow stem cell transplantation for the treatment of type 2 diabetes mellitus. *Chinese Medical Journal*.

